# Molecular docking analysis of candidate compoundsderived from medicinal plants with type 2 diabetes mellitus targets

**DOI:** 10.6026/97320630015179

**Published:** 2019-03-15

**Authors:** Pratistha Singh, Vinay Kumar Singh, Anil Kumar Singh

**Affiliations:** 1 Department of Dravyaguna, Faculty of Ayurveda, Institute of Medical Sciences, Banaras Hindu University, Varanasi, India; 2 Centre for Bioinformatics, School of Biotechnology, Institute of Science, Banaras Hindu University, Varanasi, India

**Keywords:** Diabetes mellitus, in silico docking, aldose reductase, insulin receptor, SIRT-6, medicinal plants

## Abstract

Herbal drugs are used for the treatment of diseases and disorders with its less side effects, easy availability and low cost. Several bioactive compounds have been isolated from medicinal plants such as Ficus benghelensis, Ficus racemosa, Ficus religiosa, Thespesia populena and Ficus lacur bouch were taken for screening. This study aimed to evaluate molecular interactions of selected diabetes mellitus (DM) targets with bioactive compounds isolated from Ficus benghelensis, Ficus racemosa, Ficus religiosa, Thespesia populena and Ficus lacur bouch. In this article, screening of the best substances as bioactive compounds is achieved by molecular docking analysis with 3 best selected DM target proteins i.e., aldose reductase (AR), Insulin Receptor (IR) and Mono-ADP ribosyltransferase-sirtuin-6 (SIRT6). In this analysis six potential bioactive compounds (gossypetin, herbacetin, kaempferol, leucoperalgonidin, leucodelphinidin and sorbifolin) were successfully identified on the basis of binding energy (>8.0 kcal/mol) and dissociation constant using YASARA. Out of six compounds, herbacetin and sorbifolin were observed as most suitable ligands for management of diabetes mellitus.

## Background

Incidence of Diabetes Mellitus (DM) is increasing every day among every population of the world. Conferring to a report by International Diabetes Federation (2011), there are 366 million people presently suffering from DM and it would up surge to 552 million till 2030. In 2000, the pervasiveness of Type 2 Diabetes Mellitus worldwide among adults was projected to be approximately 171 million [1] whereas in 2015 this number raised up to around 415 million [2]. Diabetes Mellitus is a cluster of metabolic disorder, an illness of hyperglycemia in which person grieves from disorders like failure of pancreas to produce insulin or insensitivity of cells towards insulin (insulin resistance). Diabetes Mellitus (DM) was previously called as "Non-insulin dependent diabetes mellitus" (NIDDM) [3]. Principal symptoms of DM are polyuria (recurrent urination), polydipsia (augmented thirst) and polyphagia (amplified hunger). Common explanations of Type 2 DM are lifestyle changes, obesity (defined as body mass index greater than 30), absence of physical activity, extreme body weight, deprived diet and anxiety [4]. There are numerous synthetic drugs available such as meglitinides, biguanides, sulfonylureas, thiazolidinediones, a-glucosidase inhibitors, dipeptidyl peptidase- IV inhibitors for treatment of DM [5], [34]. Today, researchers emphases primarily on finding of effective, low side effect and innocent therapeutic drugs to treat of DM [6]. Medicinal plants contain chemical groups (e.g., Phenolic acids, Flavonoids, Triterpenoids, Alkaloids and Carbohydrates) that hold strong antidiabetic properties, which can normalize blood glucose level. In traditional medicine, numerous medicinal plants were used such as Ficus benghalensis (Banyana), Ficus religiosa (Peepal), Ficus racemosa (Gular), Thespesia populnea, (Paras peepal) and Ficus lacorbuch (Pakar) that avoid difficulties in organization of Diabetes Mellitus.

There are a number of targets in the form of receptors are selected for treatment of DM such as Aldose Reductase (AR), Insulin Receptor (IR) and Sirtuin-6 or Mono-ADP ribosyltransferasesirtuin- 6 (SIRT6). Many more are still under exploring study to alleviate DM. AR (EC 1.1.1.21) is a monomeric, NADP-dependent oxidoreductase enzyme and a member of aldo-keto reductase multigene superfamily. Study presented that an upsurge in AR (aldose reductase) activity leads to an enlarged accumulation of intercellular sorbitol which outcomes in boosted complication in DM [7]. Another receptor called IR (Insulin receptor) which belongs to class of tyrosinekinase, a trans membrane receptor [8].One of the most common causes DM is inactivation of insulin receptor function [9]. IR is activated by insulin, IGF-1(Insulin-like growth factor) and IGF-II (Insulin growth factor-II) and any inequity in production or response of these factors adds to a cause in DM [10]. Mono-ADP ribosyltransferase-sirtuin-6 (SIRT6) or Sirtuin-6 is a stress receptive protein deacetylase and mono-ADP ribosyl transferase enzyme programmed by the SIRT-6 gene. SIRT-6 plays role in numerous molecular pathways such as aging, including DNA repair, telomere maintenance, glycolysis and inflammation. Sirtuin-6 is a possible therapeutic target for DM [11].

## Methodology

### Receptors:

**Figure 1:**
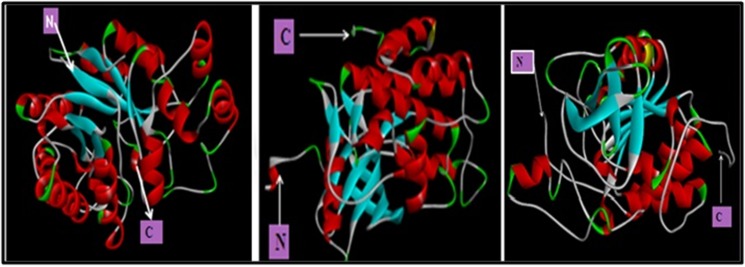
3-D Structure of AR (PDB ID: 1US0), IR (PDB ID: 1IR3) and SIRT-6 (PDB ID: 3K35) visualized by Discovery Studio 3.0

A major database CDD Conserved Domains Database in area of structural biology and computational biology for research and education [12], [13]. The three-dimensional crystal structure taken form Protein Data Bank (PDB) ie., AR (PDB ID:1US0) [14] IR (PDB ID:1IR3) [14] SIRT-6 (PDB ID: 3K35) [15] (Figure 1).

### Active site identification:

CDD BLAST [12] and Metapocket (http://projects.biotec.tudresden. de/metapocket/) server were used for identification of probable active sites. Discovery Studio 3.0 developed by Accelrys, used for visualization of three-dimensional complex structures and active site residues visualization (http://accelrys.com/).

### Ligands retrieval and assessment:

For ligand retrieval and assessment used Lipinski filter free online server services for retrieval of important molecular properties of bioactive compounds such as cLogp, hydrogen bond donors/acceptor and Molar refractivity [16]. Lipinski's rule of five were applied for selection of ligands and ADEM-TOX -Drug3 (Free ADME-Tox Tool version 3.0) used for computational prediction of Adsorption, Distribution, Metabolism, Excretion, and Toxicity properties [17].

### Docking calculation and visualization:

YASARA Autodock VINA tool, Yet another Scientific Artificial Reality Application (YASARA) was used for docking calculation. It is an online software for molecular graphics, modeling and simulation [18]. The docking analyses of potent ligands were visualized using Discovery Studio 3.0. Interactions were calculated on the basis of binding energy and containing receptor residues (Kcal/mol).

## Results and Discussion:

**Figure 2:**
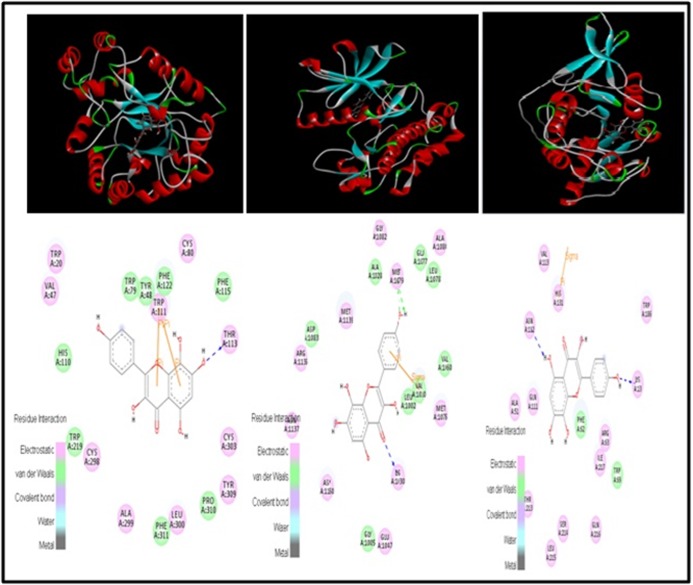
3D structure of herbacetin with AR (PDB ID: 1US0), IR (PDB ID: 1IR3) and SIRT-6 (PDB ID: 3K35)

**Figure 3:**
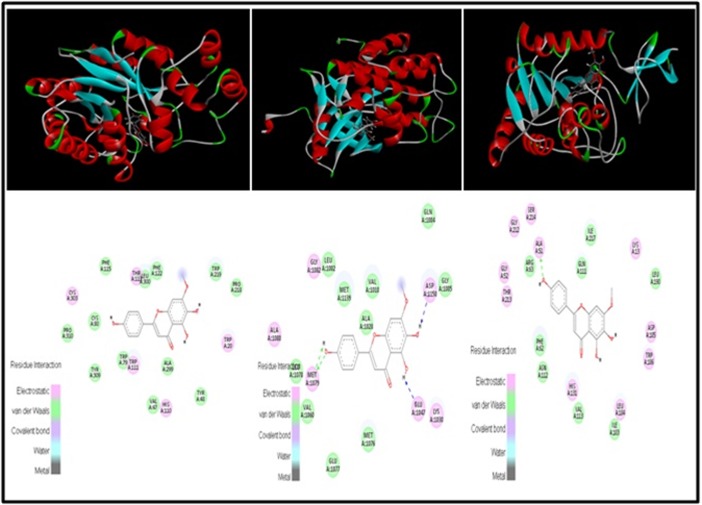
3D structure of sorbifolin with AR (PDB ID: 1US0), IR (PDB ID: 1IR3) and SIRT-6 (PDB ID: 3K35)

From five medicinal plants, 43 bioactive compound and their isolated parts (Table 1) were selected for docking calculation. All reported compounds with pubchem CID no, molecular formula, molecular weight, Conical smile (Table 2) Lipinski filter server was used to find drug likeness of selected bioactive compounds (Table 3). The anti-diabetic compounds that showed good drug likeness properties were further used for computational screening using FAF Drug server 3 (Table 4). Total selected compounds as ligand were used for docking calculation with AR, IR, SIRT-6 receptors and docking was performed by YASARA tool. Out of 43 compounds, mainly 6 compounds (Gossypetin, Herbacetin, Kaempferol, Leucodelphinidin, Leucoperalgonidin, Sorbifolin) were observed as best compounds on the basis of Energy (Table 5) Docking results obtained for each ligand with the receptor were analyzed on the basis of docking energy (Kcal/mol) and interaction of each ligand with the functional residues of AR (PDB ID:1USO), IR (PDB ID: 1IR3), SIRT-6 (PDB ID:3K35) (Figure 1) with Herbacetin and Sorbifolin respectively (Figure 2 and Figure 3). Out of six lignads Herbacetin and Sorbifolin were found best suitable ligands. In docking calculation of AR receptor and 6 ligands Trp20, Tyr48, His110, Trp111 are the most prominent binding residues (Table 6) and In case of IR, Leu1002, Met1079, Asp1150 are the most prominent binding residues with 6 ligands (Table 7) and In SIRT-6 Phe62, Gln111, Ile217, Asn112 are found to be the most prominent binding sites (Table 8). Herbacetin and Sorbifolin were observed most suitable ligands that is found in Thespesia populnea and Ficus lacor buch respectively. Leucoperalgonidin and Kaempferol were showing best docking with AR, mainly found in F. benghalensis and F. recemosa respectively.

In Ayurvedic literature, Bark of F. benghalensis, F. racemosa, F. religiosa, T. populnea and F.lacor buch are frequently known as Panchvalkala [33]. F. benghalensis is mainly found in India, Bangladesh, Sri Lanka and used to treat diarrhea, dysentery, piles, teeth disorders, rheumatism, skin disorders and diabetes. Bark of F. benghalens is has been appraised in numerous animal models by inducing diabetes using alloxan and streptozotocin. It was established that aqueous extract of bark exhibited a strong in vitro inhibitory activity against a-amylase and a-glucosidase enzymes. The ethanol extract of their leaves successfully reduced the blood glucose, triglycerides and cholesterol levels in alloxan-induced diabetic rats [19]-[22]. In the traditional systems of medicine, F. racemosa is found all over India, Northern Australia and other parts of Asia. In this plant (leaves, fruits, bark, latex, and sap of the root) are used for treatment of diabetes. Mainly bark is used for skin diseases, ulcers, diabetes, piles, dysentery, asthma, gonorrhea, leucorrhea and urinary disease. The methanol extract of bark also presented an anti-diabetic effect in Streptozotocin and alloxaninduced diabetic rats [19], [23]-[27]. F. religiosa is mainly found in the sub-himalayan tract, Bengal and central India. It has been commonly used for the treatment of various disorder such as diabetes, atherosclerosis, Alzheimer's disease, gastritis, cancer and AIDS [19], [28]-[30]. Thespesia populena from Malvaceae family has been reported to possess anti-diabetic compounds. Various experimental findings reveal that T. populnea has anti-diabetic properties. Ethanol and aqueous extract of T. populnea exhibited noteworthy anti-hyperglycemic and anti-hyperlipidemic effects on alloxan-induced diabetic rats [19], [31], [32]. Ficus lacor buch is usually known as Java fig, Pakar or Pakadi. It is found in the temperate climate of India. It is used for treatment of bleeding disorders, herpes, wound, mouth ulcers, diarrhea and leucorrhea.

## Conclusion

AR,IR,SIRT-6 used as prominent target proteins to study the interaction of selected anti-diabetic compounds isolated from various medicinal plants through the in-silico screening. A total of 6 anti diabetic compounds were selected out of 43 compounds isolated from five medicinal plants. Based on parameters like good oral bioavailability, Non-toxicity and Drug likeness Adsorption and Distribution, Metabolism, Excretion, Toxicity showing strong binding affinity with prominent binding site residues, only six compounds was selected as the best possible ligands which can be used for treatment of Type 2 Diabetes Mellitus. Leucoperalgonidin and Kaempferol were showing best docking with AR, mainly found in F. benghalensis. and F. racemosa, respectively.

## Conflict of Interest

Authors declare no conflict of interest

## Figures and Tables

**Table 1:** List of selected natural anti-diabetic compounds with plant name, common name and isolation source

S. No	Plant Name	Sources	Bioactive Compound Details	References
1	Ficus benghalensis (Banyana)	Bark	6-heptatriacontene-10-one, pentatriacontan-5-one, meso-inositol, 5,7-dimethyl ether of leucoperalgonidin- 3-0-a-L rhamnoside, 5,3-dimethyl ether of leucocyanidin, 5,7,3-trimethoxy leucodelphinidin 3-O-a-L-Rhamnoside.	[19]-[22]
2	Ficus racemosa ( Gular)	Steam, Root, Bark, Fruit.	Campesterol, Hentriacontane, Hentriacontanol,	[19], [23]-[27]
3	Ficus religiosa (Peepal)	Bark	Lupeol, Stigmasterol, Lanosterol, Campesterol. Octacosanol, Methyl oleonate, lupen-3- one. Kaempferol, Stigmasterol, Glauanol, Glauanolacetate, Esters of taraxasterol, lupeolacetate, Friedelin, Cycloartenol, Euphorbol, Hexacosanoate, Taraxerone, Tinyatoxin, Saponingluanol acetate,leucocyanidin-3-O-β-Dglucopyrancoside, Leucopelargonidin-3-O-a-L-rhamnopyranoside, Lupeol, Cerylbehenate, Lupeol acetate, a-amyrin acetate, Leucoanthocyanidin, Eucoanthocyanin, Stigmasterol.	[19], [28]-[30]
4	Thespesia populena (Paras peepal )	Bark	Herbacetin, Qurecetin, Gossypol, Populneol Calycapterin, Thespesone, Thespone, Gosypetin.	[19], [31],[32]
5	Ficus lacor buch (Pakar)	Leave, Bark	Triterpenoides, a, β amyrin, Lanosterol, Caffeic acid , Bergenin ,Compesterol, Methyl ricinolate, Scutellarein, Scutellarein, Sorbifolin, Bergapten, Bergaptol.	[33]

**Table 2:** List of selected anti-diabetic compounds and their details

S.N.	Compounds	PubChem CID	Molecular Formula	Molecular Weight (g/mol)	Canonical SMILES
1	6-heptatriacontene-10-one	56613778	C37H72O	532.982	CCCCCCCCCCCCCCCCCCCCCCCCCCCC(=O)CCC=CCCCCC
2	Meso-inositol	892	C6H12O6	180.156	C1(C(C(C(C(C1O)O)O)O)O)O
3	Pentatriacontan-5-one	54409273	C35H70O	506.944	CCCCCCCCCCCCCCCCCCCCCCCCCCCCCCC(=O)CCCC
4	Leucoperalgonidin	3286789	C15H14O6	290.271	C1=CC(=CC=C1C2C(C(C3=C(C=C(C=C3O2)O)O)O)O)O
5	Leucocyanidin	71629	C15H14O7	306.27	C1=CC(=C(C=C1C2C(C(C3=C(C=C(C=C3O2)O)O)O)O)O)O
6	Leucodelphinidin	440835	C15H14O8	322.269	C1=C(C=C(C(=C1O)O)O)[C@@H]2[C@H]([C@H](C3=C(C=C(C=C3O2)O)O)O)O
7	a-amyrin	73170	C30H50O	426.729	CC1CCC2(CCC3(C(=CCC4C3(CCC5C4(CCC(C5(C)C)O)C)C)C2C1C)C)C
8	Lupeol	259846	C30H50O	426.729	CC(=C)C1CCC2(C1C3CCC4C5(CCC(C(C5CCC4(C3(CC2)C)C)(C)C)O)C)C
9	Stigmasterol	5280794	C29H48O	412.702	CCC(C=CC(C)C1CCC2C1(CCC3C2CC=C4C3(CCC(C4)O)C)C)C(C)C
10	Lanosterol	246983	C30H50O	426.729	CC(CCC=C(C)C)C1CCC2(C1(CCC3=C2CCC4C3(CCC(C4(C)C)O)C)C)C
11	Campesterol	173183	C28H48O	400.691	CC(C)C(C)CCC(C)C1CCC2C1(CCC3C2CC=C4C3(CCC(C4)O)C)C
12	Octacosanol	68406	C28H58O	410.771	CCCCCCCCCCCCCCCCCCCCCCCCCCCCO
13	Methyl oleonate	5364509	C19H36O2	296.495	CCCCCCCCC=CCCCCCCCC(=O)OC
14	Lupen-3-one	323075	C30H48O	424.713	CC(=C)C1CCC2(C1C3CCC4C5(CCC(=O)C(C5CCC4(C3(CC2)C)C)(C)C)C)C
15	Hentriacontane	12410	C31H64	436.853	CCCCCCCCCCCCCCCCCCCCCCCCCCCCCCC
16	Hentriacontanol	68345	C31H64O	452.852	CCCCCCCCCCCCCCCCCCCCCCCCCCCCCCCO
17	Kaempferol	5280863	C15H10O6	286.239	C1=CC(=CC=C1C2=C(C(=O)C3=C(C=C(C=C3O2)O)O)O)O
18	Glauanol	101700567	C29H48O2	428.701	CC(=O)OC1CCC2(C1CCC3(C2CCC4C3(CCC5C4(CCCC5(C)C)C)C)C)C
19	Taraxasterol	115250	C30H50O	426.729	CC1C2C3CCC4C5(CCC(C(C5CCC4(C3(CCC2(CCC1=C)C)C)C)(C)C)O)C
20	Friedelin	91472	C30H50O	426.729	CC1C(=O)CCC2C1(CCC3C2(CCC4(C3(CCC5(C4CC(CC5)(C)C)C)C)C)C)C
21	Cycloartenol	92110	C30H50O	426.729	CC(CCC=C(C)C)C1CCC2(C1(CCC34C2CCC5C3(C4)CCC(C5(C)C)O)C)C
22	Euphorbol	10863111	C31H52O	440.756	CC(C)C(=C)CCC(C)C1CCC2(C1(CCC3=C2CCC4C3(CCC(C4(C)C)O)C)C)C
23	Hexacosanoate	13297142	C28H56O2	424.754	CCCCCCCCCCCCCCCCCCCCCCCCCC(=O)OCC
24	Taraxerone	92785	C30H48O	424.713	CC1(CCC2(CC=C3C4(CCC5C(C(=O)CCC5(C4CCC3(C2C1)C)C)(C)C)C)C)C
25	Tinyatoxin	442098	C36H38O8	598.692	CC1CC2(C3C4C1(C5C=C(C(=O)C5(CC(=C4)COC(=O)CC6=CC=C(C=C6)O)O)C)OC(O3)(O2)CC7=CC=CC=C7)C(=C)C
26	Lupeolacetate	92157	C32H52O2	468.766	CC(=C)C1CCC2(C1C3CCC4C5(CCC(C(C5CCC4(C3(CC2)C)C)(C)C)OC(=O)C)C)C
27	Leucoanthocyanidin	124037363	C15H14O3	242.274	C1=CC=C(C=C1)C2C(C(C3=CC=CC=C3O2)O)O
28	Herbacetin	5280544	C15H10O7	302.238	C1=CC(=CC=C1C2=C(C(=O)C3=C(O2)C(=C(C=C3O)O)O)O)O
29	Gossypol	3503	C30H30O8	518.562	CC1=C(C(=C2C(=C1)C(=C(C(=C2C=O)O)O)C(C)C)O)C3=C(C=C4C(=C3O)C(=C(C(=C4C(C)C)O)O)C=O)C
30	Populneol	2775187	C15H14O3	242.274	CC(=O)C1=C(C=CC=C1OCC2=CC=CC=C2)O
31	Calycapterin	10429470	C19H18O8	374.345	COC1=C(C2=C(C(=C1OC)OC)OC(=C(C2=O)OC)C3=CC=C(=C(C=C3)O)O
32	Thespesone	5321934	C15H14O4	258.273	CC1COC2=C1C3=C(C(=C2)C)C(=O)C(=O)C(=C3O)C
33	Thespone	5321935	C15H12O3	240.258	CC1=CC2=C(C(=CC3=C2C(=CO3)C)C)C(=O)C1=O
34	Gossypitin	5280647	C15H10O8	318.237	C1=CC(=C(C=C1C2=C(C(=O)C3=C(O2)C(=C(C=C3O)O)O)O)O)O
35	Triterpenoides	451674	C30H48O7S	552.767	CC1(CCC2(CCC3(C(=CCC4C3(CCC5C4(CCC(C5(C)COS(=O)(=O)O)O)C)C)C2C1)C)C(=O)O)C
36	β- amyrin	73145	C30H50O	426.729	CC1(CCC2(CCC3(C(=CCC4C3(CCC5C4(CCC(C5(C)C)O)C)C)C2C1)C)C)C
37	Bergenin	66065	C14H16O9	328.273	COC1=C(C=C2C(=C1O)C3C(C(C(C(O3)CO)O)O)OC2=O)O
38	Caffeic acid	689043	C9H8O4	180.159	C1=CC(=C(C=C1C=CC(=O)O)O)O
39	Methyl ricinolate	5354133	C19H36O3	312.494	CCCCCCC(CC=CCCCCCCCC(=O)OC)O
40	Scutellarein	185617	C21H18O12	462.363	C1=CC(=CC=C1C2=CC(=O)C3=C(C(=C(C=C3O2)OC4C(C(C(C(O4)C(=O)O)O)O)O)O)O)O
41	Sorbifolin	3084390	C16H12O6	300.266	COC1=C(C(=C2C(=C1)OC(=CC2=O)C3=CC=C(C=C3)O)O)O
42	Bergapten	2355	C12H8O4	216.192	COC1=C2C=CC(=O)OC2=CC3=C1C=CO3
43	Bergaptol	5280371	C11H6O4	202.165	C1=CC(=O)OC2=CC3=C(C=CO3)C(=C21)O

**Table 3:** Drug Likeness using Lipinski's rule

S. No.	Compounds	Molecular mass less than 500	Hydrogen bond donor less than 5 hydrogen bond donors	Hydrogen bond acceptor less than 10 hydrogen bond acceptors	LOGP High lipophilicity expressed as log P less than 5	Molar refractivity less should be between 40-130	Status
1	6-heptatriacontene-10-one	532	0	1	13.634	173.239	Not accepted
2	Pentatriacontan-5-one	506	0	1	13.078	164.099	Not accepted
3	Meso-inositol	180	6	6	-3.835	36.041	Not accepted
4	Leucoperalgonidin	290	5	6	1.331	72.214	Accepted
5	Leucocyanidin	306	6	7	1.037	73.879	Not accepted
6	leucodelphinidin	322	7	8	0.743	75.543	Accepted
7	a-amyrin	428	1	1	8.105	130.674	Not accepted
8	Lupeol	426	1	1	8.025	130.649	Not accepted
9	Stigmasterol	412	1	1	7.8	128.123	Not accepted
10	Lanosterol	426	1	1	8.479	132.879	Not accepted
11	Octacosanol	410	1	1	10.141	132.802	Not accepted
12	Methyl oleonate	296	0	2	6.197	91.467	Not accepted
13	Lupen-3- one	312	5	6	-0.053	77.146	Not accepted
14	Campesterol	400	1	1	7.635	123.599	Not accepted
15	Hentriacontane	436	0	0	12.339	145.241	Not accepted
16	Hentriacontanol	452	1	1	11.311	146.653	Not accepted
17	Kaempferol	286	4	6	2.305	72.386	Accepted
18	Glauanol	428	0	2	7.793	126.509	Accepted
19	Esters of taraxasterol	428	1	1	8.105	130.674	Not Accepted
20	Lupeolacetate	468	0	2	8.596	140.197	Not accepted
21	Friedelin	426	0	1	8.457	129.744	Not accepted
22	Cycloartenol	426	1	1	8.169	130.719	Not accepted
23	Euphorbol	440	1	1	8.725	137.426	Not accepted
24	Hexacosanoate	424	0	2	9.932	133.115	Not accepted
25	Taraxerone	424	0	1	8.377	129.719	Not accepted
26	Tinyatoxin	598	2	8	4.736	160.141	Not accepted
27	Leucoanthocyanidin	242	2	3	2.215	67.219	Accepted
28	Herbacetin	302	5	40	2.011	74.05	Accepted
29	Gossypol	518	6	8	3.846	139.167	Not accepted
30	Populneol	242	1	3	3.174	3.174	Accepted
31	Calycapterin	374	2	8	2.714	94.757	Accepted
32	Thespesone	257	0	4	2.141	68.63	Accepted
33	Thespone	240	0	3	3.218	68.676	Accepted
34	Gossypetin	318	6	8	1.716	75.715	Accepted
35	Triterpenoides	550	1	7	5.437	140.072	Not accepted
36	β amyrin	426	1	1	8.169	130.719	Not accepted
37	Bergenin	328	5	9	-1.201	72.24	Not accepted
38	Caffeic acid	179	2	4	-0.139	43.812	Not accepted
39	Methyl ricinolate	312	1	3	5.168	92.858	Accepted
40	Scutellarein	461	6	12	-1.644	103.13	Not accepted
41	Sorbifolin	300	3	6	2.428	77.366	Accepted
42	Bergapten	216	0	4	2.373	57.435	Accepted
43	Bergaptnol	202	1	4	2.071	52.548	Accepted

**Table 4:** FAF Drug Results: Best selected compounds on the basis of adsorption, distribution, metabolism, excretion and toxicity.

S. N.	Compound Name	Heavy atom	Hetero atom	Solubility (mg/L)	Oral (Bioavailability) (EGAN)	Oral (Bioavailability) (VEBER)	Ratio (H/C)	Mar-75	Status
1	Leucoperalgonidin	21	6	30803.51	Good	Good	0.4	Good	Accepted
2	Leucodelphinidin	23	8	444470.47	Good	Good	0.53	Good	Accepted
3	Kaempferol	21	6	12543.68	Good	Good	0.4	Good	Accepted
4	Leucoanthocyanidin	18	3	17228.74	Good	Good	0.2	Warning	Accepted
5	Herbacetin	22	7	10239.43	Good	Good	0.46	Good	Accepted
6	Populneol	18	3	7959.6	Good	Good	0.2	Bad	Accepted
7	Calycapterin	27	8	6459.43	Good	Good	0.42	Warning	Accepted
8	Thespesone	19	4	15936.11	Good	Good	0.27	Warning	Accepted
9	Thespone	18	3	7740.16	Good	Good	0.2	Warning	Accepted
10	Gossypetin	23	15	12386.97	Good	Good	0.53	Good	Accepted
11	Methyl ricinolate	22	3	3645.68	Good	Good	0.16	Bad	Accepted
12	Sorbifolin	22	6	6706.61	Good	Good	0.38	Good	Accepted
13	Bergapten	16	4	14084.11	Good	Good	0.33	Warning	Accepted
14	Bergaptnol	15	4	15635.88	Good	Good	0.36	Warning	Accepted

**Table 5:** YASARA Docking Calculation: Binding Energy (Kcal/mol) of receptors and ligands complexes.

S.N.	Compound Name (CID NO.)	AR	IR	SIRT-6
1	Gossypetin(5280647)	8.006	8.429	8.569
2	Herbacetin (5280544)	9.623	8.165	8.632
3	Kaempferol (5280863)	10.034	7.881	8.533
4	Leucodelphinidin (440835)	8.012	7.915	8.234
5	Leucoperalgonidin (3286789)	9.02	7.756	7.874
6	Sorbifolin (3084390)	9.391	8.063	8.697

**Table 6:** Interacted, Reported, Predicted active site residues of AR and compounds.

S.No.	Compound Name	Interacted Residues	Reported Active Site Residues	Predicted Active Site Residues	Common Residues
1	Gossypetin	Trp20,Val47,Tyr48,Gln49,Trp79,His110,Trp111,Phe115,Phe122,Phe115,Phe122,Trp219,Cys298,Ala299,Leu300,Cys303,Tyr309,Phe311	Gly18,Thr19,Trp20,Ile35,Tyr48,Lys202,His110,Trp111,Ser159,Asn160,Gln183,Tyr209,Ser210,Pro211,Leu212,Gly213,Ser214,Ala208,Ile260,Pro261,Lys262,Ser263,Glu271,Asn272,Phe273	Trp20,Lys21,Pro218,Trp219,Trp79,Cys80,Trp111,Thr113,Phe115,Phe122,Leu300,Cys303,Tyr309,Ala299,Cys298,His110,Val47,Tyr48Asn160,Tyr209,Ser159,Gln183,Ser210,Lys77,ASP43,Ile260,Thr19,Gly18,Lys262,Ser214,Pro211,Asp216,Leu212,Pro215Pro261,Leu228,Arg268,Ser263,Asn272,Ala245,Glu271,Thr243,Thr244,Glu229,Ser226,Val264,Thr265,Val297,Ser302,Leu124,Leu301Phe311,Pro310,Gln49,Phe121His46,Leu108,Val130,Gly213,Glu267,Asn50,Ser22	Trp20,Tyr48,His110,Trp111
2	Herbacetin	Trp20,Val47,Tyr48,Trp79,Cys80,His110,Trp111Thr113,Phe115,Phe122,Trp219,Cys298,Ala299,Leu300,Cys303,Tyr309,Pro310,Phe311			Trp20,Tyr48,His110,Trp111
3	Kaempferol,	Trp20,Val47,Tyr48,Trp79,Cys80,His110,Trp111,Thr113,Phe115,Phe122,Trp219,Cys298,Ala299,Leu300,Cys303,Tyr309,Pro310,Phe311			Trp20,Tyr48,His110,Trp111
4	Leucodelphinidin	Trp20,Val47,Tyr48,Gln49,Lys77,Trp79,His110,Trp111,Phe122,Asn160,Gln183,Tyr209,Ser210,Trp219,Trp20,Val47,Tyr48,Trp79,Cys80,His110,Trp111,Thr113Phe115,Phe122,Trp219,Cys298,Ala299,Leu300,Cys303,Tyr309,Pro310,Phe311Cys298,Leu300			Trp20,Tyr48,His110,Trp111
5	Leucoperalgonidin	Trp20,Val47,Tyr48,Trp79,Cys80,His110,Trp111,Thr113,Phe115,Phe122,Trp219,Cys298,Ala299,Leu300,Cys303,Tyr309,Phe311			TRP20,Tyr48,His110,Trp111
6	Sorbifolin	Trp20,Val47,Tyr48,Trp79,Cys80,His110,Trp111,Thr113,Phe115,Phe122,Pro218,Trp219,Ala299,Leu300,Cys303,Tyr309,Pro310			TRP20,Tyr48,His110,Trp111

**Table 7:** Interacted, Reported, Predicted active site residues of IR and compounds.

S. N.	Compound name	Interacted Residues	Reported Active Site Residues	Predicted Active Site Residues	Common Residues
1	Gossypetin	Leu1002,Gly1005Val1010,Ala1028,Lys1030,Glu1047,Val1060,Met1076,Glu1077,Leu1078Met1079,Gly1082,Asp1083,Arg1136Asn1137,Met1139,Asp1150	Gly1005,Val1010,Ala1028,Thr1031,Glu1077,Met1079,Asp1083,Asp1132,Arg1136,Asn1137,Asp1150,Lys1168,Gly1167,Lys1168,Gly1169,Leu1117,Pro1172,Leu1181,Lys1182,Gly1184,Agn1215	Arg1039,Ile1042,Thr1160,Arg1164,Asp1161,Glu1043,Arg1155,Asn1046,Asp1156,Ile1157,Glu1040,Arg1041,Gly1152,Lys1165,Gly1166,Val1185,Lys1168,Gly1167,Thr1154,Arg1131,Val1129Phe1186,Gly1169,Met1153,Thr1187,Leu1170,Asn1124Lys1127,Ser1037,Phe1007,Leu1171,Gly1184Thr1188Lys1251Lys1030Phe1044,Glu1047,Ala1048,Met1051Val1074,Met1076,Ser1006,Glu1179,Ser1189,Asp1183,Pro1250,Asp1150,Pro1172,Phe1151,Asp1132,Val1060Gly1149,Gly1005,Val1010,Asn1249,Asn1137,Gln1004,Ala1028Gly1008,Arg1136Glu1077,Met1139,Gly1003,Leu1078,Met1079,Leu1002Val1173,Asp1083,Ala1080,Gly1082,His1081,Ser1086,Lys1085,Arg1000,Glu1001Glu1012,Tyr1087,Asn1097,Ser1090,Pro1099,Leu1133,Ala1134Trp1175,Ser1194,Arg1174,Asn1215,Gln1208,His1130	Gly1005,Asn1137,Leu1002,Asp1150,Asp1083,Asn1137,Met1079,Arg1136
2	Herbacetin	Leu1002,Gly1005,Val1010,Ala1028,Lys1030,Glu1047,Val1060,Met1076,Glu1077,Leu107Met1079,Ala1080Gly1082,Asp1083,Arg1136,Asn1137,Met1139,Asp1150			Gly1005,Asn1137,Leu1002,Asp1150,Asp1083,Asn1137,Met1079,Arg1136
3	Kaempferol	Leu1002,Val1010,Ala1028,Lys1030,Glu1047,Val1060,Met1076,Glu1077,Leu1078,Met1079,Ala1080,Gly1082,Asp1083,Asn1137,Met1139,Asp1150			Asn1137,Leu1002,Asp1150,Asp1083,Asn1137,Met1079
4	Leucodelphinidin	Leu1002,Gly1003,Gly1005,Ser1006,Val1010,Ala1028,Lys1030,Met1076,Glu1077,Leu1078,Met1079,Gly1082Asp1083,Arg1136,Asn1137,Met1139,Asp1150			Gly1005,Asn1137,Leu1002,Asp1150,Asp1083,Met1079,Arg1136
5	Leucoperalgonidin	Leu1002,Gly1003Val1010,Ala1028,Lys1030,Glu1047,Val1060,Met1076,Glu1077,Leu1078,Met1079,Ala1080,Gly1082,Asp1083,Met1139,Gly1149,Asp1150,Phe1151			Leu1002,Asp1150
6	Sorbifolin	Leu1002,Gln1004,Gly1005,Val1010,Ala1028,Lys1030,Glu1047,Val1060,Met1076,Glu1077,Leu1078,Met1079,Ala1080,Gly1082,Met1139,Asp1150			Leu1002,Gly1105,Met1079,Asp1150

**Table 8:** Interacted, Reported, Predicted active site residues of 3k35 and compounds.

S.N	Compound name	Interacted Residues	Reported Active Site Residues	Predicted Active Site Residues	Common Residues
1	Gossypetin	Lys13,Gly50,Ala51,Phe62,Arg63,Trp69,Gln111,Asn112,Val113,His131,Trp186,Leu190,Gly212,Thr213,Ser214,Ile217	Gly52,Ser54,Thr55,Phe62,Arg63,His93,Gln111,Asn112,Asp114,Gly212,Ile217,Leu239,Gln240,Gly254,Tyr255	Val68,Trp69,Glu72,Pro78,Phe62,Trp186,Met155,Asp185Leu184,His66,Met71,Lys79,Ala77,Pro60,Phe80,Lys13,Arg63,Gly156,Ile217,Glu187,Gly67,Ile183,Asp81,Gly64,Pro65,Asp61,Asp188,Glu20,Phe84,Met134,Arg218,Gly58,Ile59,Thr55,Gln240,Lys15,Ser214,Val113,Gln216,His131Ser189,Leu190,Leu18,Leu239,Ala51,Pro219,Asn238,Thr213,Leu215,Gln111,Tyr255,Pro241,Gly52,Gly212,Ser220,Ser57,Ala56,Phe22,Asp114,Asn112,Gly50,Gly221,Ser54,Gly115Thr49Gly254,Val237,Val256Ile53Asp257,Thr90,Glu,258	Phe62,Gln111,Asn112,Gly212,Ile217,
2	Herbacetin	Lys13,Gly50,Ala51,Phe62,Arg63,Trp69,Gln111,Asn112,Val113,His131,Trp186,Thr213,Ser214,Leu215,Gln216,Ile217			Phe62,Gln111,Asn112,Ile217,
3	Kaempferol	Lys13,Gly50,Ala51,Gly52,Phe62,Arg63,Trp69,Gln111,Asn112,His131,Trp186,Gly212,Thr213,Ser214,Leu215,Gln216,Ile217			Phe62,Gln111,Asn112,Gly212,Ile217,
4	Leucodelphinidin	Gly50,Ala51,Phe62,Arg63,Trp69,Gln111,Asn112,His131,Leu184,Asp185,Trp186,Asp188,Thr213,Ile217			Phe62,Gln111,Asn112,Ile217
5	Leucoperalgonidin	Gly50,Ala51,Phe62,Arg63,Trp69,Gln111,Asn112,His131,Leu184,Asp185,Trp186,Asp188,Leu190,Thr213,Ile217			Phe62,Gln111,Asn112,Ile217
6	Sorbifolin	Lys13,Gly50,Ala51,Gly52,Phe62,Arg63,Gln111,Asn112,Val113,His131,Ile183,Leu184,Asp185,Trp186,Leu190,Gly212,Thr213,Ser214,Ile217			Phe62,Gln111,Asn112,Gly212,Ile217
